# The Irish Medical Schools

**Published:** 1896-09-05

**Authors:** 


### The Irish Medical Schools.

### MEDICAL STUDY IN IRELAND.

In Ireland the seats of medical education are Dublin, Belfast, Cork, and Galway.

The Dublin University, of worldwide fame under the name of Trinity College, Dublin, has a School of Physic formed by amalgamation of its school with that of the King and Queen's College of Physicians, and, in regard to clinical teaching, working in connection with a considerable number of the Dublin hospitals. Fees for lectures and hospital practice, £122 6s. 6d.

The Catholic University School of Medicine. Total cost, including degree fees for Royal University of Ireland, £163 1s.; for conjoint examination R.C.P. and S.I., including examination fees, £162 15s.

The Schools of Surgery in connection with the Royal College of Surgeons of Ireland form a complete school, which includes the Carmichael and Sedwick Schools of Medicine: Fees for lectures and hospital practice for the conjoint diploma, R.C.S. and P.I., £121 5s. Total cost, including licence fees, £161 5s.

Both at Belfast, Cork, and Galway there are medical schools in connection with the Queen's Colleges established in those towns.

